# The Genetic Basis of Psoriasis

**DOI:** 10.3390/ijms18122526

**Published:** 2017-11-25

**Authors:** Francesca Capon

**Affiliations:** Department of Medical and Molecular Genetics, School of Basic and Biomedical Sciences, King’s College London, London SE1 9RT, UK; francesca.capon@kcl.ac.uk

**Keywords:** psoriasis, genome-wide association studies, genome-wide association studies, GWAS, Th17 activation, IL-36

## Abstract

Psoriasis is widely regarded as a multifactorial condition which is caused by the interaction between inherited susceptibility alleles and environmental triggers. In the last decade, technological advances have enabled substantial progress in the understanding of disease genetics. Genome-wide association studies have identified more than 60 disease susceptibility regions, highlighting the pathogenic involvement of genes related to Th17 cell activation. This pathway has now been targeted by a new generation of biologics that have shown great efficacy in clinical trials. At the same time, the study of rare variants of psoriasis has identified interleukin (IL)-36 cytokines as important amplifiers of Th17 signaling and promising targets for therapeutic intervention. Here, we review these exciting discoveries, which highlight the translational potential of genetic studies.

## 1. The Multifactorial Inheritance of Psoriasis

While the role of environmental triggers (e.g., stress, mechanical trauma and streptococcal infections) is well documented in psoriasis, epidemiological studies have repeatedly demonstrated that this condition has an important genetic component. Familial recurrence is well documented and disease concordance is higher in monozygotic vs. dizygotic twins [1,2]. Thus, psoriasis is widely regarded as a multifactorial disorder caused by the interaction between inherited susceptibility alleles and environmental risk factors [3].

## 2. The Conflicting Results of Linkage Studies

The first studies that sought to illuminate the genetic architecture of psoriasis were based on linkage analysis, a statistical approach that enables the localization of disease genes to well-defined chromosomal regions. While linkage studies proved extremely successful in mapping loci underlying monogenic diseases, it quickly became apparent that these methods were not suited to the analysis of multifactorial conditions. In the case of psoriasis, linkage analysis identified nine different regions (known as Psoriasis Susceptibility (PSORS)1-9) that were supposed to contribute to disease susceptibility [4]. Of these, only PSORS1 was robustly validated in all examined cohorts [4]. This led researchers to conclude that the interval harbored a major genetic determinant for the disease. Weaker linkage signals at the PSORS2 [5,6] and PSORS4 [7,8] regions were observed in more than one dataset, suggesting that these were genuine susceptibility loci. Linkage to the remaining PSORS regions (PSORS-3, -5, -6, -7, -8, -9) could not be replicated in independent studies [4].

## 3. PSORS1, the Major Genetic Determinant of Psoriasis

The PSORS1 locus maps to the Major Histocompatibility Complex (MHC) on chromosome 6p21 [9]. Once linkage to this region was conclusively validated, fine mapping studies were carried out by various groups [9,10]. These investigations defined a consensus 150 kb minimal interval spanning the MHC class I region and encompassing nine genes [9,10]. Of these, three (*HLA-C*, *CCHCR1* and *CDSN*) were highly polymorphic and harbored coding variants that were significantly associated with psoriasis [11]. *HLA-C* encodes a MHC class I receptor that participates to immune responses through the presentation of antigens to CD8^+^ T lymphocytes. It is therefore a very plausible candidate gene, especially as serological studies carried out as early as the mid-1970s had identified an association between psoriasis and the HLA-Cw6 allele [12]. While the function of *CCHCR1* is still poorly understood, *CDSN* encodes a keratinocyte protein involved in skin desquamation, a process that is known to be altered in psoriasis [13]. While the above observations argued for a role of *CDSN* variation in the pathogenesis of psoriasis, subsequent studies have shown that the association with *CDSN* alleles is likely to be a secondary one, reflecting linkage disequilibrium (i.e., co-inheritance) with HLA-Cw6 [10,14].

In recent years, several studies have sought to dissect the mechanisms whereby HLA-Cw6 contributes to the molecular pathogenesis of psoriasis. Given the role of HLA-C in antigen presentation, it has been hypothesized that HLA-Cw6 may have a high binding affinity for one or more psoriasis autoantigens. Indeed, Arakawa et al have found that HLA-Cw6 can present a specific melanocyte auto-antigen (ADAMTS-like protein 5) to CD8^+^ T cells [15]. Structural biology studies have also shown that the peptide binding grove of HLA-Cw6 has high-affinity for LL-37 [16], a molecule that has been described as a T-cell autoantigen in psoriasis [17]. As the T cell infiltrate observed in psoriatic lesions is highly polyclonal [18], other molecules that are preferentially recognized by HLA-Cw6 are likely to emerge in the coming years.

As the entire *HLA-C* gene region is extremely polymorphic, disease associated alleles have also been identified in regulatory regions [19]. Such variants may also contribute to disease pathogenesis by modifying the expression of HLA-Cw6.

## 4. Additional MHC Associations

Given its very prominent position in the genetic landscape of psoriasis, the MHC region has been the subject of very detailed genetic studies, carried out in European and Asian populations. These have identified many additional association signals that are independent of HLA-Cw6 and map to *HLA-A*, *HLA-B* and *HLA-DQA1* [20,21,22]. While these findings have been validated in independent studies, their mechanistic basis has yet to be investigated.

## 5. The PSORS2 Locus

The PSORS2 region was initially identified through the linkage analysis of a multi-generation north-American pedigree where psoriasis was inherited as an autosomal dominant condition [6]. Linkage to the same region was subsequently documented in an extended Asian kindred [23]. With the advent of next-generation sequencing, mutations in the *CARD14* gene were identified as the molecular defect underlying disease segregation in these two families [24]. Of note, *CARD14* mutations have also been observed in two conditions that are phenotypically related to plaque psoriasis, namely pityriasis rubra pilaris [25] and generalised pustular psoriasis [26,27].

*CARD14* encodes an adaptor protein that is highly expressed in keratinocytes, where it mediates TRAF2-dependent NF-κB signal transduction [28]. The *CARD14* mutations that have been characterised so far have proved to be gain-of-function alleles, which cause constitutive NF-κB activation, leading to enhanced production of pro-inflammatory cytokines [24,25,26].

Interestingly, genome-wide studies (see below) have identified common *CARD14* SNPs that are associated with psoriasis in case-control datasets [29]. Thus, *CARD14* harbors rare and highly-penetrant mutations that segregate in multiplex pedigrees, as well as common susceptibility alleles of small effect.

As *CARD14* mutations are only found in a small number of psoriasis kindreds [26], additional genes responsible for monogenic forms of the disease remain to be identified.

## 6. The PSORS4 Locus

The PSORS4 region maps to chromosome 1q21, where it spans the Epidermal Differentiation Cluster (EDC) [7]. This is an evolutionary conserved genomic segment, harboring more than 60 genes involved in terminal keratinocyte differentiation [30]. Subsequent studies have shown that the deletion of two EDC genes (*LCE3B* and *LCE3C*, encoding two late cornified envelope proteins) is strongly associated with psoriasis [31,32], suggesting a pathogenic role for the disruption of skin barrier function.

## 7. The Advent of Genome-Wide Association Studies

The early 2000s witnessed important advances in the effort to catalogue human genetic variation and in the development of high-throughput genotyping technologies. This progress enabled the implementation of genome-wide association studies (GWAS), where large case-control datasets were typed at tens of thousands of Single Nucleotide Polymorphisms (SNPs). The first psoriasis GWAS was undertaken in 2007, based on the genotyping of 25,125 SNPs in 466 cases and 500 controls [33]. Several additional studies were carried out in subsequent years [14,34,35,36]. As the establishment of multi-national consortia enabled the ascertainment of larger resources, samples sizes grew at a steady pace, with the latest published GWAS reporting the analysis of 19,000 cases and 280,000 controls [37]. Advances in genotyping technologies and the design of methods for imputation (the process whereby the genotype of a SNP can be inferred from that of neighboring markers) also enabled the analysis of larger numbers of SNPs (up to 9 million per study).

The success of GWAS (see below) spurred the design of targeted genotyping platforms, such as the ImmunoChip (which specifically enabled the analysis of SNPs that had previously been associated with immune mediated disorders) and the Exome chip (which only focused on markers mapping to coding regions).

## 8. Pathogenic Insights Afforded by Large-Scale Association Studies

Studies carried out on genome-wide [14,33,34,35,36], exome-wide [38] and Immunochip [29,37] genotyping platforms have now identified a total of 63 psoriasis susceptibility loci (47 uncovered in GWAS, 15 in studies based on the ImmunoChip and 1 in an exome-wide survey). Only a small number of these genomic segments span a single gene, with the majority encompassing multiple transcripts and some mapping to gene deserts. While this has undoubtedly complicated the identification of the underlying susceptibility genes, likely candidates have been uncovered for many associated regions, based on one or more of the following criteria: (i) direct functional characterization of the lead SNP [39]; (ii) linkage disequilibrium of the lead SNP with a deleterious variant in the candidate gene [29]; (iii) localization of the lead SNP within an enhancer regulating the expression of the candidate gene [37], (iv) evidence that the lead SNP is associated with increased/decreased expression of the candidate gene [40].

Importantly, the candidate genes identified so far tend to cluster to a small number of immune pathways. These include antigen presentation (*HLA-C* and *ERAP1*), innate antiviral signaling (*IFIH1*, *DDX58*, *TYK2*, *RNF114*) and most notably, Th17 cell activation. The latter is the process whereby naïve CD4^+^ T lymphocytes differentiate into IL-17 producing Th17 cells. The pathogenic nature of abnormal Th17 signaling is underscored by the identification of psoriasis associated alleles within *IL12B* and *IL23A* [35] (encoding the two subunits of IL-23, a cytokine that drives the polarization of T lymphocytes towards the Th17 lineage), *IL23R* [33] (encoding the IL-23 receptor), *TRAF3IP2* [34] (encoding an adaptor molecule driving NF-κB signal transduction downstream of IL-17) and *NFKBIZ* [41] (a target of IL-17 signaling in keratinocytes).

In keeping with the pro-inflammatory role of the IL-23/Th17 axis, biologics that block the activation of this pathway have proved to be effective therapeutics [42]. While ustekinumab (Stelara), a molecule that blocks the *IL12B* gene product, has been in use in the clinic since 2009, the development of IL-23A and IL-17 inhibitors was informed by the results of GWAS and is therefore more recent [43]. Secukinumab (Cosentyx), ixekizumab (Taltz) and broadalumab (Kyntheum) are IL-17 blockers that have been approved for the treatment of moderate to severe plaque psoriasis, in the US and Europe. Likewise, the IL-23 inhibitor Guselkumab (Tremfya) has received US Food and Drug Administration (FDA) approval, with other IL-23 antagonists (tildrakizumab and risankizumab) generating promising results in clinical trials [44] (Table 1). Interestingly, IL-23 is also down-regulated by some small molecules that are approved for the treatment of psoriasis (e.g., the phosphodiesterase 4 inhibitor apremilast (Otezla) and the fumaric acid ester dimethylfumarate (Skilarence)). Likewise, IL-17 production appears to be inhibited by the traditional Chinese medicine ingredient Indigo naturalis. Thus, the pathogenic insights obtained in large-scale association studies have identified IL-23 and IL-17 as key disease drivers that can be targeted by various classes of therapeutics.

## 9. Beyond GWAS

Despite their success, GWAS suffer from some limitations that are intrinsic to the design of genotyping chips. These were mostly devised to detect association with common SNPs and therefore cannot be applied to the analysis of copy-number variants (CNV) (i.e., large insertion/deletions or duplications) and rare sequence changes. While rare alleles of large effect do not appear to play a significant role in the pathogenesis of psoriasis [38,45], CNV have been repeatedly implicated in disease susceptibility. Besides the *LCE3B/LCE3C* deletion detected at the PSORS4 locus [31], psoriasis is associated with an increased β-defensin copy number [46]. The latter result was obtained in a study that specifically focused on β-defensin genes, based on their anti-microbial and pro-inflammatory function. Thus, novel genetic determinants of psoriasis susceptibility could be uncovered by analysing further CNV that span plausible candidate genes.

## 10. Rare Clinical Variants of Psoriasis Have a Distinctive Genetic Basis

In the last few years, the advent of whole-exome sequencing (the process whereby all coding sequences of a genome can be sequenced in one experiment) has enabled the identification of hundreds of genes causing rare diseases. In the field of psoriasis, this technology has allowed researchers to shed new lights on pustular variants of the disease. These are a group of rare and severe conditions, which are not associated with HLA-Cw6 [47] and show limited response to the biologics used to good effect in common plaque psoriasis [48].

Two breakthrough studies published in 2011 identified mutations of the *IL36RN* gene in patients affected by generalised pustular psoriasis, a condition that presents with flares of skin pustulation and systemic upset [49,50]. *IL36RN* encodes a molecule that modulates the pro-inflammatory activity of IL-36 cytokines, by binding their receptor and blocking downstream signal transduction. Loss-of-function mutations of the *IL36RN* gene abolish this inhibitory activity, causing uncontrolled IL-36 signaling [49,50].

While *IL36RN* disease alleles are found in approximately 25% of affected individuals [51], mutations in a second pustular psoriasis gene (*AP1S3*) have been identified in an additional 10% of patients [52]. Of note, *AP1S3* defects have also been linked to excessive IL-36 production, indicating that the disruption of IL-36 homeostasis is a key disease driver [53].

Importantly, pustular forms of psoriasis often present with concurrent plaque psoriasis [54], which suggested that abnormal IL-36 signaling may also contribute to the common form of the disease. Indeed, a recent study has demonstrated the presence of an IL-36 signature among the genes that are up-regulated in plaque psoriasis. The same authors also found that IL-36 can amplify the effects of Th17 activation and that IL-36 blockade has significant anti-inflammatory effects on psoriatic skin lesions [55]. Thus, the genetic analysis of rare variants of psoriasis has identified IL-36 signalling as a novel therapeutic target for plaque and pustular forms of psoriasis.

## 11. Conclusions and Outlook

Genetic studies have now uncovered more than 60 psoriasis susceptibility loci. Not all the underlying genes have been conclusively identified, so that fine mapping studies and functional characterization of disease alleles are still required. Thanks to the advent of imputation methods, it is possible to derive the genotypes of dense marker maps across disease associated regions. This will enable the identification of candidate susceptibility alleles, which can then be characterized in pathologically relevant cell types. As a wealth of information is now available on the function of non-coding regions, combining the results of the above genetic analyses with the characterization of tissue-specific regulatory elements would be particularly effective in the discovery of causal susceptibility variants.

At the same time, genetic approaches are being applied to the study of other aspects of the disease. For instance, candidate gene studies have investigated whether *HLA-C* and *IL23R* alleles can influence treatment outcome. While these investigations have documented an association between HLA-Cw6 and response to ustekinumab [56], the results were obtained in small datasets, so that the analysis of larger samples is required. Studies also need to be expanded beyond candidate genes and encompass the analysis of sequence variants on a genome-wide scale.

Genotype data is now becoming available for prospectively ascertained population cohorts such as those recruited by UK Biobank (500,000 volunteers who have provided a detailed medical history and are being monitored over the years [57]) and 23andMe (270,000 individuals who provided their medical history when requesting direct to consumer genetic testing [58]). The individuals enrolled in these population-based surveys are likely suffer from milder disease than those ascertained in hospital departments and investigated in GWAS. The analysis of these new datasets could show that some of the sequence variants that are associated with psoriasis in GWAS do not confer disease risk among patients recruited in population cohorts. This would offer important insights into the genetic determinants of disease severity.

Finally, the availability of longitudinal data is now allowing the implementation of GWAS investigating the link between genetic variation and disease course. The latter approach has recently been applied to Crohn’s disease, leading to the identification of four loci that influence clinical prognosis [59].

Thus, future GWAS hold the promise to identify biomarkers of drug response and disease course, paving the way for the personalized management of psoriasis.

## Figures and Tables

**Table 1:** Biologics targeting the products of psoriasis susceptibility genes.

Name (Trade Name)	Target (Genetic Association)	Development Stage
Ustekinumab (Stelara)	p40 subunit shared by IL-12 and IL-23 (SNPs in *IL12B*, which encodes p40, are associated with psoriasis susceptibility [14,33])	Approved for the treatment of moderate to severe psoriasis
Secukinumab (Cosentyx)	IL-17A (No risk alleles in *IL17A*, but psoriasis associated SNPs have been identified in *TRAF3IP2*, which encodes an IL-17 receptor adaptor [14,34])	Approved for the treatment of moderate to severe psoriasis
Ixekizumab (Taltz)	IL-17A (No risk alleles in *IL17A*, but psoriasis associated SNPs have been identified in *TRAF3IP2*, which encodes an IL-17 receptor adaptor [14,34])	Approved for the treatment of moderate to severe psoriasis
Broadalumab (Kyntheum)	IL-17 receptor subunit A (IL-17RA) (No risk alleles in *IL17RA*, but psoriasis associated SNPs have been identified in *TRAF3IP2*, which encodes an IL-17 receptor adaptor [14,34])	Approved for the treatment of moderate to severe psoriasis
Guselkumab (Tremfya)	p19 subunit unique to IL-23 (SNPs in *IL23A*, which encodes p19, are associated with psoriasis susceptibility [14,35])	Approved for the treatment of moderate to severe psoriasis
Tildrakizumab	p19 subunit unique to IL-23 (SNPs in *IL23A*, which encodes p19, are associated with psoriasis susceptibility [14,35])	Phase III trial successfully completed [60]
Risankizumab	p19 subunit unique to IL-23 (SNPs in *IL23A*, which encodes p19, are associated with psoriasis susceptibility [14,35])	Phase II trial successfully completed [61]
